# Self-assembly of highly conductive self-n-doped fullerene ammonium halides and their application in the *in situ* solution-processable fabrication of working electrodes for alcohol electrooxidation†

**DOI:** 10.1039/c8ra00100f

**Published:** 2018-03-06

**Authors:** H. H. Wang, X. Sun, Z. C. Lin, Z. F. Pang, X. Q. Kong, M. Lei, Y. F. Li

**Affiliations:** Department of Chemistry, Zhejiang University Hangzhou 310027 China leiming@zju.edu.cn; CAS Research/Education Center for Excellence in Molecular Sciences, Institute of Chemistry, Chinese Academy of Sciences Beijing 100190 China

## Abstract

Stable and highly conductive self-n-doped fullerene ammonium halides are promising optoelectronic materials. It is necessary to thoroughly understand their structure–function relationship and to develop their applications. Here, the assembly behaviors of the self-n-doped fullerene ammonium halides, as well as the functional areas in the well-developed 2D–3D lamellar structures in their ordered aggregates are systematically characterized using comprehensive methods. In the self-assembly, the solvation effect of DMSO promotes the flexibility of side-chains and drives the formation of fullerene ammonium halides into ordered bilayer structures. The conductivity-active area, which contains tightly packed halide anions sandwiched between fullerenes, provides good electron transfer property. Remarkably, residual DMSO in the side-chain area can induce aqueous Pd precursor into the highly conductive framework. After reduction, Pd nanoparticles are immobilized in the confined spaces within the conductive support. The resulting electrode can be used to electrooxidize ethanol. This study provides a facile solution strategy for the *in situ* fabrication of electrocatalysts on working electrodes, which can be applied in direct alcohol fuel cells.

## Abstract 2

Self-assembled highly conductive fullerene ammonium halides are potential support of metal-based nanoparticles toward alcohol electrooxidation.

## Graphical abstract

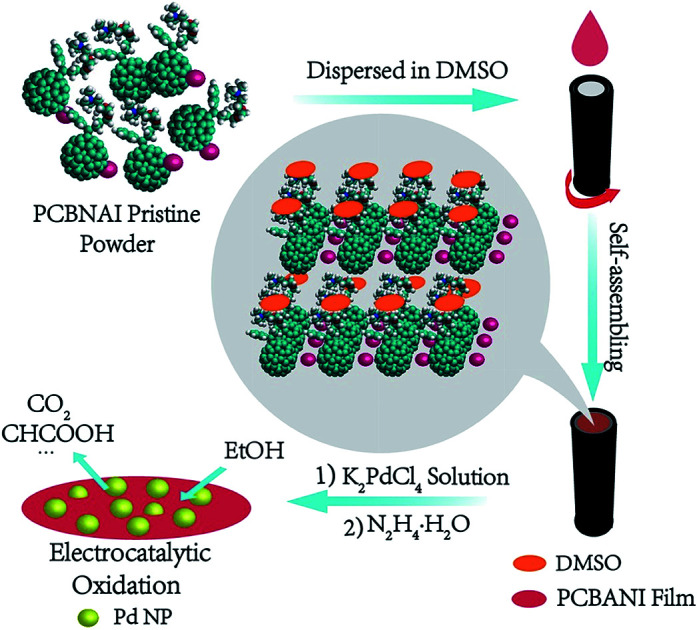

## Introduction

**Scheme 1:**
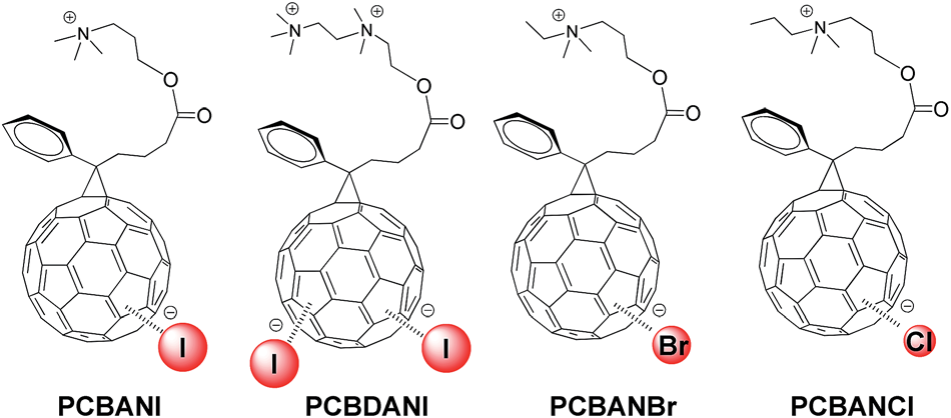
Structures of self-n-doped fullerene ammonium halides.

Stable and highly conductive self-n-doped fullerene ammonium halides are promising cathode interfacial layer materials that can facilitate electron transfer and improve the power conversion efficiencies of organic solar cells.^1,2^ Through systematic studies using molecular models of fullerene ammonium iodide (PCBANI) (Scheme 1), we elucidated the mechanism of a cross-self-n-doping process that involves strong anion–π noncovalent interactions between iodide anions and core fullerenes.^3^ We found that the doping effect in PCBANI caused the separation of cation–anion pairs and increased polarization. Thus, PCBANIs instinctively aggregated to achieve stability. Moreover, we previously reported that PCBANI self-assembled into a layered stacking supramolecular system through the delicate balance of iodide anion–C_60_ π, electrostatic, and C_60_ π–π interactions.^4^ The proposed ET model, in which the iodide sandwiched in the fullerene core acts as a shuttle to transfer electrons *via* intramolecular/intermolecular synergistic redox processes, shows that the ordered and tightly packed fullerenes that sandwich iodide can facilitate electron transfer along the network system.^3,4^

We recently performed systematic studies on halide anion–fullerene π interactions in self-n-doped fullerene ammonium halides containing I^−^, Br^−^, and Cl^−^ and demonstrated the universality of the halide anion (I^−^, Br^−^, and Cl^−^) *n*-doping of fullerene (Scheme 1).^5^ Device experiments revealed that the conductivities of the self-n-doped fullerene ammonium halides successively decreased in the order of PCBANI, PCBANBr and PCBANCl. This indicated that the size of the halide anion is a crucial factor in determining the electron transport property of these materials. In addition, functions of the side-chain needed to be studied. To develop novel functions of these highly conductive fullerenes, it is necessary to thoroughly understand their self-assembly behaviour and detect the functional areas within their supramolecular structures.

In terms of the anode catalysts employed in direct alcohol fuel cells (DAFCs), the alternative palladium (Pd)-based nanocatalysts have attracted much attention because Pd is less expensive and shows greater resistance to CO poisoning than Pt.^6,7^ In the recent years, considerable efforts have been devoted to the development of Pd-based nanoparticles (NPs), which are dispersed on various supports for electrocatalytic alcohol oxidation.^8–11^ The supports include conductive polymers,^12^ carbon nanotubes,^13^ carbon fibers,^14^ graphene oxide^15^ and graphene,^16^ where polycarbon-based materials are commonly used. Unfortunately, the strong planar π–π stacking of 2D graphene sheets results in their reversible agglomeration^17,18^ and the drastic loss of electroactive sites during electrode assembly. Consequently, porous 3D graphene^19–22^ has emerged as an alternative support material for catalyst loading to improve mass transfer and maximize the accessibility of the catalyst surface. By contrast, 0D fullerenes (C_60_) are less studied supports with relatively low conductivities due to their polycrystallization or short-range order structures.^23–26^ Notably, the development of single-crystalline, 1D-phototreated C_60_ nanorods with high aspect ratios and increased electron mobilities has provided new perspectives on the use of fullerenes as an electrocatalyst support.^27^ Generally, in designing the support for the loading of a catalyst, it is crucial to transfer the complex catalyst system onto the electrode, while maintaining its activity and reproducibility. However, the majority of available cathodic catalysts for DAFCs have been limited by their complicated ex situ fabrication procedures. Therefore, the *in situ* fabrication of electrocatalysts on an electrode is desirable, economical, and has potential applications for the large-scale manufacture of DAFC electrodes. The introduction of a synergetic functionalized support is of vital importance for such a fabrication strategy.

In the present study, comparative studies on the self-assembly structures of self-n-doped fullerene ammonium halides were performed to understand the relationship between film conductivity and microstructure. Furthermore, a simple and practical method for the *in situ* fabrication of electrocatalysts on working electrodes was facilitated by the unique self-assembly structure of highly conductive self-n-doped fullerene films. The immobilized Pd NPs catalyst system was designed on the basis of the following considerations: (1) the 2D–3D self-n-doped fullerene framework had relatively high electron conductivity (1 to 2 s m^−1^). At the same time, strong polar functional groups enhanced the adhesive strength of the support to the electrode preventing its dissociation during operation. (2) The solvation effect of residual DMSO on the side-chain area within the framework facilitated the diffusion of the Pd precursor into the area of the ionic group adjacent to fullerene. (3) Given the advantages of the microstructure effect and weak interactions, the aggregation of ultrafine Pd NPs could be prevented by immobilizing Pd in the confined spaces within packed fullerenes along the side-chain area. In this study, we demonstrated that the highly conductive self-assembled fullerene film can act as a template and support for the immobilization of Pd NPs. Furthermore, it provides a facile solution method for the *in situ* fabrication of fuel cell electrodes for ethanol electrooxidation.

## Results and discussion

### Comparative studies on the self-assembly structures

#### XRD analysis

**Fig. 1:**
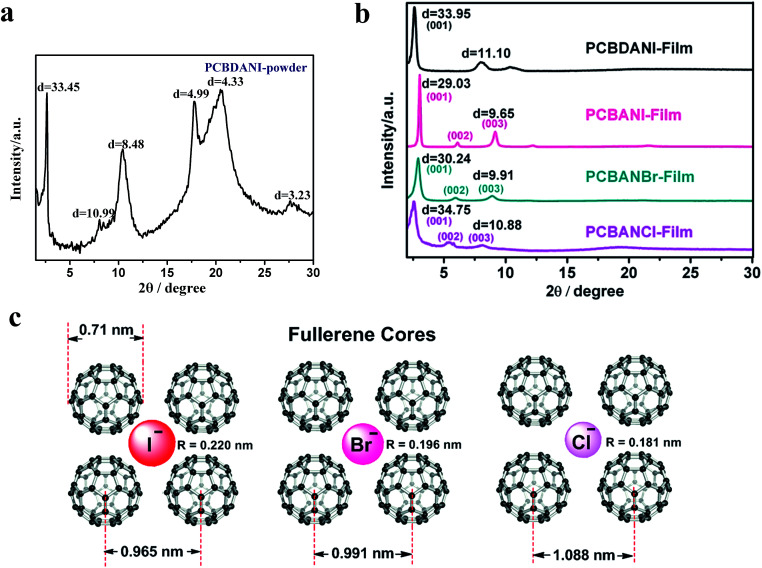
(a) XRD pattern of pristine powder PCBDANI. (b) XRD patterns of self-assembled PCBDANI, PCBANBr, and PCBANCl in comparison with that of PCBANI. (c) Schematic diagram illustrating the structure and size of self-assembled PCBANI, PCBANBr and PCBANCl (side-chains are omitted) based on the Z-view image of the optimized structure of self-assembled PCBANI obtained by computational modeling (the fullerene has a diameter of 0.71 nm (ref. 29)).

To investigate the factors of iodide number and side-chain length, we comprehensively characterized self-assembled films of PCBDANI, which is a homologue of PCBANI, in accordance with the established methodology. PCBDANI pristine powder was obtained from the methylation of its precursor (PCBDAN)^1^ in chloroform after centrifugation and decanting. As shown in Fig. 1a, XRD analysis revealed a peak at 2*θ* ≈ 2.5° with a *d*-spacing value of 3.345 nm, which corresponded to the length of a bilayer structure. The presence of broad peaks and this result indicate the existence of short-range ordered arrangements in the disordered aggregates. The spin-coated film from the DMSO dispersion of the above pristine powder exhibited a well-defined XRD pattern. This result could be attributed to the Bragg reflections from the (001) plane with a lamellar periodicity *d*-spacing value of 3.395 nm (Fig. 1b), which corresponded to the length of a bilayer. Moreover, a broad peak at 2*θ* ≈ 8.0°, which corresponded to 1.110 nm distance, was presumably caused by the neighboring fullerene cores.^28^ Therefore, the XRD analysis provided evidence for the ordered multi-bilayer alignment of PCBDANI. Compared with that in the XRD spectrum of PCBANI (Fig. 1b), the slightly broader peak with a shift and larger *d*-spacing value in the XRD spectrum of PCBDANI may be attributed to the increase in the side-chain length and number of iodide molecules. Additional iodides were sandwiched in the crowded space between fullerene cores, thus decreasing the orderliness of arrangement governed by the iodide–π and fullerene π–π interactions. This phenomenon could explain the absence of the (001) and (002) reflections. Although XRD results indicated that PCBDANI exhibited poorer orderliness of arrangement than PCBANI, it still possessed slightly higher conductivity (1.98 S m^−1^*vs.* 1.50 S m^−1^),^2^ indicating that the tight packing of iodide in fullerene cores was the dominant factor of conductivity.

Then, we investigated the XRD patterns of PCBANBr and PCBANCl films fabricated through DMSO dispersion. The well-defined XRD patterns of PCBANBr and PCBANCl (Fig. 1b) were attributed to the Bragg reflections from the (001) to (003) planes. Broad peaks at 2*θ* ≈ 2.5° were assigned to reflections from the (001) plane and had *d*-spacing values of 3.024 and 3.475 nm for PCBANBr and PCBANCl, respectively; these values corresponded to those of bilayer lengths and were consistent with those of the increased side-chain lengths. Similar to that in PCBANI, the neighboring fullerene core peaks in the XRD spectra of PCBANBr and PCBANCl overlapped with the (003) plane peak and corresponded to 0.991 and 1.088 nm distance, respectively. The increased distance between the fullerene core and the size reduction of halide anions (*R*_I_ − [0.220 nm] > *R*_Br_ − [0.196 nm] > *R*_Cl_ − [0.181 nm]) suggested that the assembly into fine structures was governed by the balance between iodide–π and fullerene π–π interactions. We constructed a schematic of the structural sizes of fullerene ammonium halides on the basis of the Z-view image of the optimized self-assembled PCBANI structure obtained by computational modelling (Fig. 1c). It suggested that small anions cannot be tightly sandwiched between fullerenes, resulting in loose self-assembled structures and decreased electron transport properties.

#### SEM and TEM analysis

**Fig. 2:**
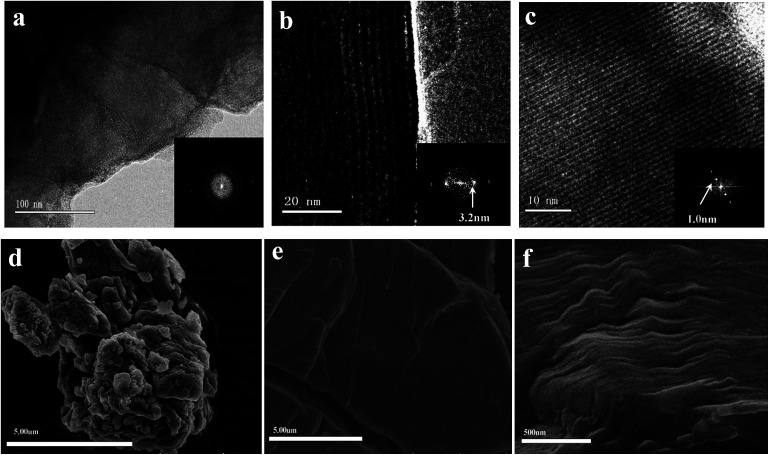
HR-TEM image of PCBDANI (a) pristine powder with disordered structure. (b), (c) Cross-sectional images of self-assembled PCBDANI with lamellar structures and the corresponding FFT analysis; the periodicities of the lamellae are 3.2 nm and 1.0 nm, respectively. A large-area image of the lamellae with 3.2 nm periodicity is shown in Fig. S1.† (d) FESEM images of PCBDANI pristine powder with disordered array; (e), (f) self-assembled PCBDANI appears to be solid and tightly packed.

To further understand the architecture of the assembly, we dispersed the above PCBDANI samples in ethanol through extensive sonication. Afterward, we subjected the samples to high-resolution transmission electron microscopy (HR-TEM) analysis for bright-field imaging. As shown in Fig. 2a, the image and the corresponding fast Fourier transform (FFT) analyses showed that the pristine powder existed as a disordered aggregate, whereas the cross-sectional HR-TEM image revealed well-developed lamellar structures in the abovementioned self-assembled sample (Fig. 2b and c). FFT analysis of the lamellar parts of the TEM images yielded periodicities of 3.2 and 1.0 nm. These values were in agreement with the values of bilayer thickness and space between neighboring fullerene cores obtained from the XRD analysis. The distance between the adjacent fullerenes (1.0 nm) suggested a strong C_60_ π–π interaction^28^ and a highly ordered alignment of the fullerene cores. Moreover, field-emission scanning electron microscopic (FESEM) images showed that disordered aggregates were formed in the PCBDANI pristine powder (Fig. 2d), whereas large areas of the self-assembled PCBDANI film appeared to be in the solid state (Fig. 2e and f).

#### X-ray photoelectron spectroscopy analysis

**Fig. 3:**
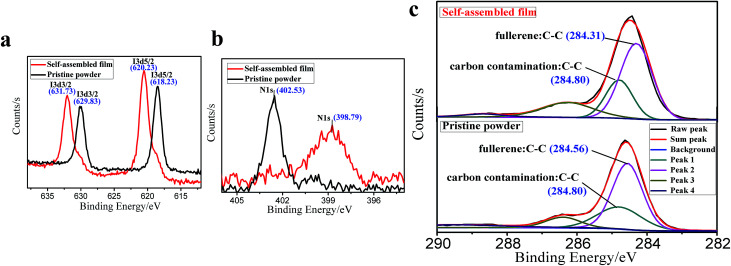
Comparative XPS spectra of PCBDANI's pristine powder and its self-assembled film. (a) I 3d3/2 and I 3d5/2; (b) N 1s; and (c) C 1s. The binding energy scale was calibrated using the C 1s line (284.8 eV) from the carbon contamination.

To clarify the interactions that govern the film assembly, we probed the electronic structure of the related elements in PCBDANI pristine powder and self-assembled film through X-ray photoelectron spectroscopy (XPS). Fig. 3a shows that the binding energy of iodide in the self-assembled PCBDANI film increased by 1.90 and 2.00 eV for the I 3d3/2 and I 3d5/2 peaks, respectively, relative to that in the pristine powder. Meanwhile, the binding energy decreased by 3.74 eV for N 1s peaks and 0.25 eV for C 1s peaks in the XPS spectrum of the self-assembled PCBDANI film compared with that of the pristine powder (Fig. 3b and c). These changes in the electronic structure of the relevant elements implied that self-assembly occurred through a balance among iodide C_60_ π–π, iodide anion–π, and anion–cation electrostatic interactions in intra- and intermolecular aspects and resulted in the tight packing of PCBDANIs. However, the arrangement orderliness of PCBDANI was not as good as that of PCBANI, which could be attributed to increased amounts of iodide.

#### Solid-state nuclear magnetic resonance study on the morphology of the side-chain

**Fig. 4:**
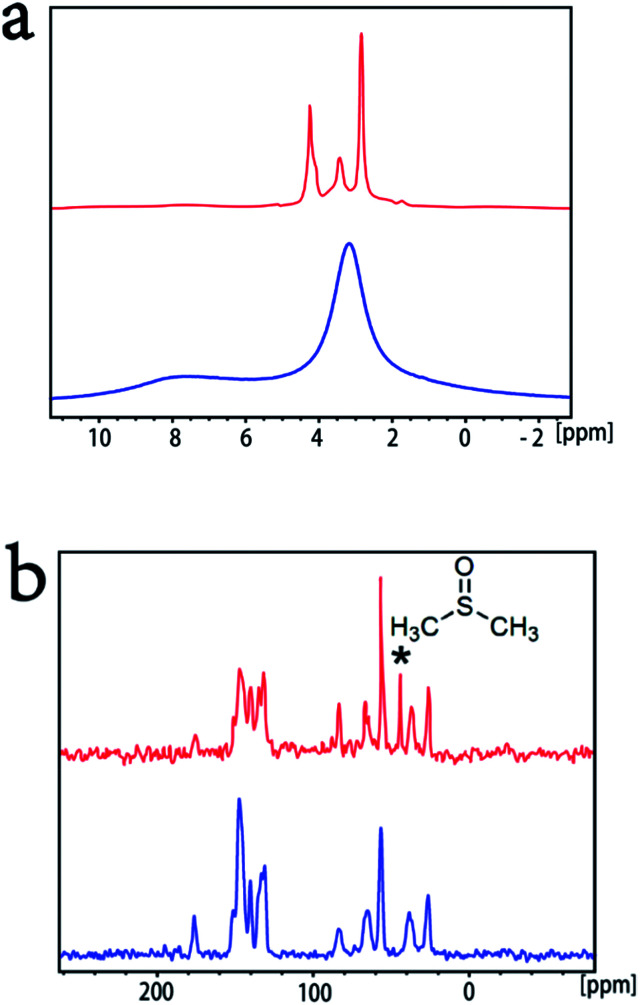
Comparative solid-state nuclear magnetic resonance (ssNMR) study on PCBANI's pristine powder and its self-assembled film. (a) ^1^H single pulse and (b) ^13^C CP spectra of self-assembled film (red) and pristine powder (blue); the peak at approximately 42 ppm marked with star is assigned to the methyl signal of DMSO. All spectra are referenced using adamantine as a secondary reference against TMS.

To characterize the side-chain morphology of self-n-doped fullerene ammonium halide aggregates, we performed a solid-state nuclear magnetic resonance (ssNMR)^30,31^ study on PCBANI's pristine powder and the self-assembled film spin-coated from its DMSO dispersion. The ^1^H and ^13^C CP spectra of the self-assembled film (red line) and the pristine powder (blue line) are shown in Fig. 4a and b, respectively. The peak at approximately 42 ppm of the self-assembled film sample (red line) in the ^13^C spectrum was assigned to the methyl group of the residual DMSO. The narrowed ^1^H peaks of the self-assembled film suggested that the side-chains were highly dynamic. This result indicated DMSO's role in the dispersion of the PCBANI pristine power. Thus, we believe that in the self-assembled film, the solvation effect of DMSO promoted the flexibility of side-chains and drove the formation of PCBANIs into ordered bilayer structures and highly conductive frameworks. In this framework, the residual DMSO was adjacent to the side-chain and acted as a template for the introduction of the aqueous catalyst precursor solution into the confined space within the side-chain area. After transformation, the conductive substrate-supported nanoparticles were obtained and were used as electrocatalysts.

#### Functional area in self-assembled self-n-doped fullerene ammonium halides

**Fig. 5:**
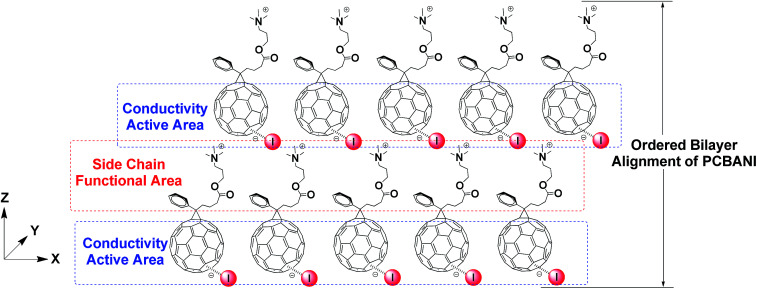
Functional area in self-assembled self-n-doped PCBANI.

We can detect the functional area in the self-assembled structures of the self-n-doped fullerene ammonium halides on the basis of the abovementioned studies. The area that consists of the fullerene core and halide anion is the conductivity-active area. Side-chain is another factor that governs the self-assembly and properties of the self-n-doped fullerene ammonium halides. Fig. 5 shows that in the lamellar structure, the ammonium-cation terminated side-chains access iodides in the upper PCBANI layer to form electrostatic interactions, which can form a 2D–3D structure and reinforce the network of electron transport. Moreover, the side-chain functional area is filled with polar groups that can form an electrostatic interaction field. Therefore, the confined spaces between the flexible side-chains are most likely used to disperse and immobilize NP hybrids, such as metal catalysts, to exhibit the synergistic function.

### Application in solution-processable fabrication of working electrodes for alcohol electrooxidation

#### Immobilization of Pd nanocatalysts on self-n-doped fullerene ammonium iodides

**Fig. 6:**
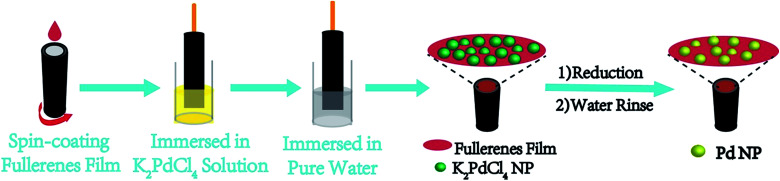
Procedure for *in situ* fabrication of an immobilized Pd nanoparticle electrocatalyst on a working electrode using PCBANI and PCBDANI as conductive supports.

We devised a three-step all-solution process to immobilize Pd NPs on a working electrode *in situ* with PCBANI and PCBDANI as conductive supports. The process was devised on the basis of the understanding of the self-assembly structure of the highly conductive self-n-doped fullerene ammonium halides. As shown in Fig. 6, self-assembled PCBANI and PCBDANI films were first spin-coated from their DMSO dispersions on a glassy carbon disc electrode. As far as measurement and characterization are concerned, parallel fabrication was conducted on a glassy carbon flake with the same shape and size. Then, electrodes were treated with an aqueous K_2_PdCl_4_ precursor solution. Subsequently, electrodes anchored with the Pd precursor were treated with hydrazine hydrate solution. After rinsing, the electrocatalytic activity of the obtained working electrodes was evaluated.

**Fig. 7:**
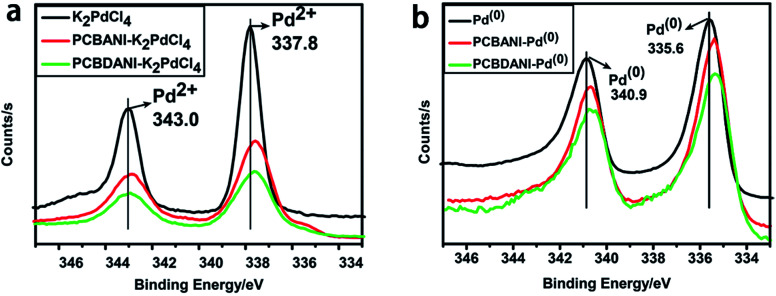
XPS measurements of Pd^2+^ and Pd^0^ on the surface of PCBANI and PCBDANI films.

**Fig. 8:**
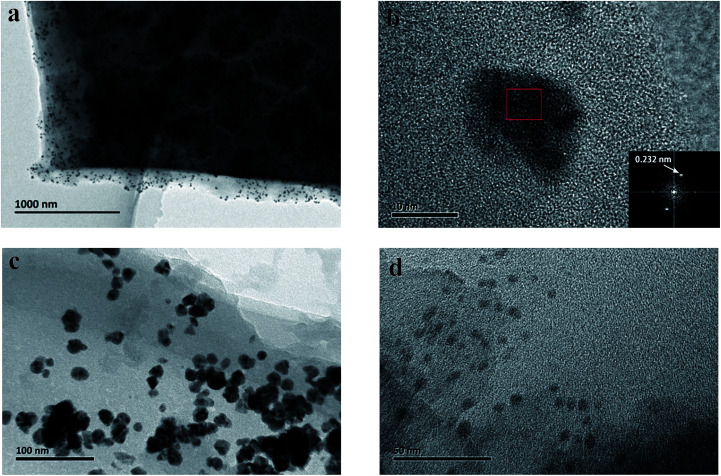
HR-TEM image of Pd nanoparticles immobilized on self-assembled PCBANI (a, b) and PCBDANI films (c, d).

We carefully characterized the immobilized Pd^2+^ precursor and Pd^0^ NPs during fabrication to demonstrate our design concept. The XPS measurements shown in Fig. 7 indicated the existence of Pd^2+^ and Pd^0^ on the surfaces of PCBANI and PCBDANI films. Slight systematic shifts (∼0.2 eV) in the binding energies of PCBANI and PCBDANI films were compared with those of the control samples. The results suggested that the Pd element and support weakly interacted. As expected, residual DMSO had a delicate role in inducing and locating aqueous-based K_2_PdCl_4_ in the framework. Driven by the affinity of water for DMSO, K_2_PdCl_4_ diffused and dispersed into an ionic group area and was then immobilized through multiple interactions. After treatment with the hydrazine hydrate solution, Pd NPs with sizes of 5–10 nm were obtained as revealed through HR-TEM (Fig. 8). As shown in Fig. 8b, FFT analysis further revealed the coexistence of a lattice spacing of 0.232 nm in Pd NP. This spacing corresponded to the (111) interplanar distance of a standard Pd crystalline lattice. The dispersibility of Pd NPs on the PCBANI film was better than that on the PCBDANI film. This result can be attributed to the highly ordered microstructure of the self-assembled PCBANI as observed through XRD analysis.

#### Electrooxidation of ethanol

**Fig. 9:**
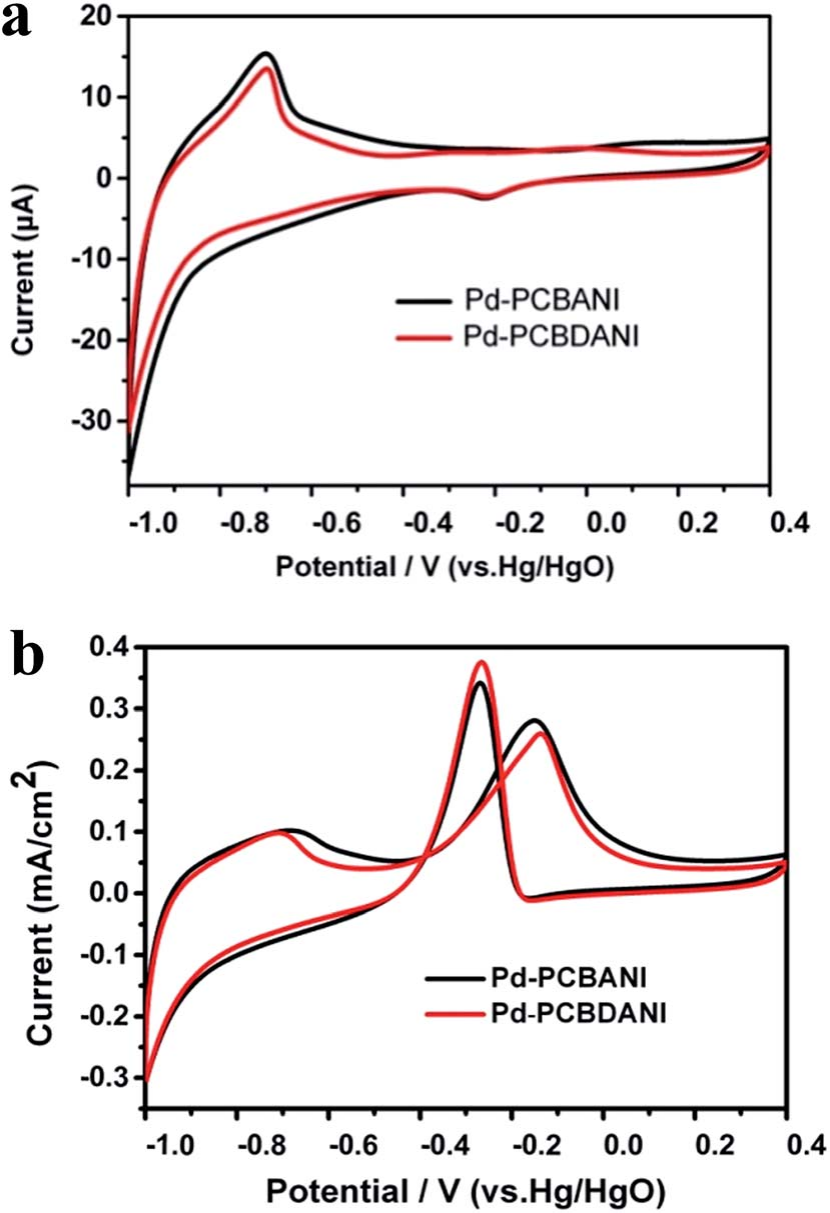
CV results of Pd-PCBANI (black line) and Pd-PCBDANI (red line) coated electrode in blank solution containing 1 M KOH (a) and in 1 M KOH containing 0.5 M ethanol (b). Scan rate: 0.05 V s^−1^.

Direct ethanol fuel cells are renewable and environmentally friendly energy sources that have attracted considerable research interest given their unique advantages, such as high energy density (*W*_e_ = 8.0 kW h kg^−1^) and easier storage and transport.^10,11^ Therefore, using the as-fabricated working electrode, we performed preliminary CV measurements to evaluate the electrocatalytic activity of ethanol oxidation in a basic medium. The three modified electrodes exhibited a classic reduction peak in the potential region from −0.1 to −0.3 V (Fig. 9a). This result could be ascribed to the electroreduction of Pd oxide. The peak potentials of Pd reduction were 0.270 and 0.261 V for Pd-PCBANI and Pd-PCBDANI, respectively. As shown in Fig. 9b, the onset potentials of ethanol oxidation with Pd-PCBANI and Pd-PCBDANI electrodes were approximately −0.46 and −0.44 V, respectively. Furthermore, the normalized current density of the ethanol oxidation peak of the Pd-PCBDANI electrode was 0.38 mA cm^−2^, which was slightly higher than that of the Pd-PCBANI electrode (0.34 mA cm^−2^). The above results indicated that the as-fabricated working electrode showed electrocatalytic activity toward ethanol oxidation.

Thus, we developed a facile method for the *in situ* fabrication of electrocatalysts. The interaction between the Pd precursor and conductive support was essential for the design of a highly dispersed composite nanocatalyst. At the same time, preventing the irreversible aggregation of ultrafine Pd NPs was crucial to maintain catalytic activity and durability. Although the catalytic activity of the working electrode is lower than that of the present supported Pd catalyst, it can be improved by optimizing operation parameters and conditions such as film quality, precursor concentration, and treatment time. Moreover, the solution fabrication of new catalyst systems with low Pd usage has potential applications in the *in situ* fabrication of large-area electrodes for electrocatalysis given its simplicity, improved reproducibility, and low cost.

## Experimental

### Materials

All chemicals were purchased from Aldrich Chemical Co. and Acros Chemical Co. and were used without further purification unless stated otherwise.

#### Fabrication of the films of self-assembled self-n-doped fullerene ammonium halides

Glass substrates were cleaned sequentially with detergent aqueous solution, acetone, deionized water, and absolute ethyl alcohol each for 10 min. After drying, the DMSO dispersion of fullerene ammonium halides (200 mg mL^−1^) was directly spin-coated onto the substrates at 3000 rpm for 3 min.

#### X-ray diffraction (XRD)

XRD patterns were measured at room temperature using a RIGAKU D/MAX 2550/PC X-ray diffractometer with monochromatic Cu Kα radiation (*λ* = 1.54184 Å) at 18 kW.

#### Scanning electron microscopy (SEM)

The morphologies of the films were examined with a HITACHI SU -8010 SEM at 1–10 kV. Glass was used as a substrate and platinum coating was obtained using a MCI1000 ion sputter.

#### High-resolution transmission electron microscopy (HR-TEM)

HR-TEM images were obtained with a JEM-2100F microscope. One drop of absolute ethyl alcohol of the sample was deposited on a carbon-coated copper grid (230 mesh). The excess solution on the grid was drained off with a filter paper, left to dry and then, observation was performed at room temperature of 200 kV (HR-TEM). The HR-TEM image of the edge-on lamellar morphology was observed from a microtome section (50 nm thick) conducted on an ultramicrotome (Leica EM UC7).

#### X-ray photoelectron spectroscopy (XPS)

XPS measurements of the samples were carried out using a Thermo Scientific ESCALAB 250Xi spectrometer. All spectra were obtained using a monochromatic Al Kα (1486.8 eV) X-ray radiation at 180 W. The typical operating pressure was 2 × 10^−7^ Pa. The binding energy scale was calibrated using the C 1s line (284.6 eV) from the carbon contamination.

#### Solid-state NMR (ssNMR)

All the solid-state NMR spectra were obtained on a Bruker Avance III HD 400 MHz spectrometer operating at a resonance frequency of 400.13 MHz for ^1^H and 100.62 MHz for ^13^C using MAS probes equipped with 3.2 mm spinner modules at a spinning speed of 15 KHz. Cross-polarization (CP) contact time was 2 ms. The magic angle and field homogeneity of the spectrometer were optimized using KBr and adamantine at room temperature, respectively. In addition, all spectra were referenced using adamantine as a secondary reference against TMS.

#### Preparation of immobilized Pd nanoparticles on a working electrode

In a glove box maintaining constant 22% RH, 1 mg ml^−1^ PCBANI or PCBDANI DMSO dispersion was applied to the surface of the glassy carbon disc electrode, which was cleaned sequentially in acetone, deionized water, and ethanol under sonication for 5 min. Then, the self-assembled fullerene films were obtained by spin-coating at a rotation speed of 100 rpm for 2 h. Next, the electrodes coated with fullerene films were immersed sequentially in 1 mg ml^−1^ K_2_PdCl_4_ aqueous solution for 30 min and then in pure water for 10 min. Subsequently, the electrodes anchored with the Pd precursor were treated with hydrazine hydrate solution for 5 min. After rinsing, the obtained working electrodes could be used for electrocatalytic activity evaluation.

#### Electrocatalytic activity evaluation

The electrocatalytic performances of PCBANI and PCBDANI-supported Pd NPs were compared by the cyclic voltammetry (CV) method with a three-electrode cell at room temperature. A Pt wire and an Hg/HgO electrode were used as the counter and reference electrodes, respectively, and a glassy carbon electrode coated with Pd NPs was used as the working electrode. A KOH aqueous solution (1 M) was used as the supporting electrolyte during the electrochemical measurement. Before the electrochemical measurement was carried out, the electrolyte solution was bubbled with nitrogen for at least 30 min to remove the dissolved oxygen.

## Conclusions

In summary, the self-assembly structures of highly conductive self-n-doped fullerene ammonium halides were systematically characterized, and the functional areas in the well-developed 2D–3D lamellar structures in their ordered aggregates were illustrated. The active area, which involves halide anions sandwiched between tightly packed fullerenes, conferred good electron transfer property to the self-n-doped fullerene ammonium halides. Combined with the template functionality of the side-chain, the highly conductive framework could immobilize Pd NPs through interactions between the metal precursor and conductive support. The resulting electrode can be used to electrooxidize ethanol. This study provides an all-solution strategy for the *in situ* fabrication of electrocatalysts. Moreover, the proposed strategy has vast potential applications in the large-area printing of fuel cell electrodes.

## Conflicts of interest

There are no conflicts to declare.

## Supplementary Material

RA-008-C8RA00100F-s001

## References

[cit1] Li S. S., Lei M., Lv M. L., Watkins S. E., Tan Z. A., Zhu J., Hou J. H., Chen X. W., Li Y. F. (2013). Adv. Energy Mater..

[cit2] Jiao W. X., Ma D., Lv M. L., Chen W. W., Wang H. Q., Zhu J., Lei M., Chen X. W. (2014). J. Mater. Chem. A.

[cit3] Chen W. W., Jiao W. X., Li D. B., Sun X., Guo X., Lei M., Wang Q., Li Y. F. (2016). Chem. Mater..

[cit4] Sun X., Chen W. W., Liang L. J., Hu W., Wang H. H., Pang Z. F., Ye Y. X., Hu X. R., Wang Q., Kong X. Q., Jin Y. Z., Lei M. (2016). Chem. Mater..

[cit5] Sun X., Ji L. Y., Chen W. W., Guo X., Wang H. H., Lei M., Wang Q., Li Y. F. (2017). J. Mater. Chem. A.

[cit6] Liu D., Guo Q. H., Hou H. Q., Niwa O., You T. Y. (2014). ACS Catal..

[cit7] Rabis A., Rodriguez P., Schmidt T. J. (2012). ACS Catal..

[cit8] Cui C. H., Yu S. H. (2013). Acc. Chem. Res..

[cit9] Akhairi M. A. F., Kamarudin S. K. (2016). Int. J. Hydrogen Energy.

[cit10] Kamarudin M. Z. F., Kamarudin S. K., Masdar M. S., Daud W. R. W. (2013). Int. J. Hydrogen Energy.

[cit11] Rao L., Jiang Y. X., Zhang B. W., You L. X., Li Z. H., Sun S. G. (2014). Prog. Chem..

[cit12] Wang A. L., Xu H., Feng J. X., Ding L. X., Tong Y. X., Li G. R. (2013). J. Am. Chem. Soc..

[cit13] Wei Y., Zhang X. Y., Luo Z. Y., Tang D., Chen C. X., Zhang T., Xie Z. L. (2017). Nano-Micro Lett..

[cit14] Pierozynski B. (2013). Int. J. Electrochem. Sci..

[cit15] Chen X. M., Wu G. H., Chen J. M., Chen X., Xie Z. X., Wang X. R. (2011). J. Am. Chem. Soc..

[cit16] Singh R. N., Awasthi R. (2011). Catal. Sci. Technol..

[cit17] Park S., Shao Y., Wan H., Rieke P., Viswanathan V., Towne S., Saraf L., Liu J., Lin Y., Wang Y. (2011). Electrochem. Commun..

[cit18] Yu D. S., Dai L. M. (2010). J. Phys. Chem. Lett..

[cit19] Chen W., Li S., Chen C., Yan L. (2011). Adv. Mater..

[cit20] Tang Z., Shen S., Zhuang J., Wang X. (2010). Angew. Chem., Int. Ed..

[cit21] Hu C. G., Cheng H. H., Zhao Y., Hu Y., Liu Y., Dai L. M., Qu L. T. (2012). Adv. Mater..

[cit22] Zhang Z. Y., Dong Y., Wang L., Wang S. (2015). Chem. Commun..

[cit23] Zhang Y., Jiang L., Li H., Fan L. Z., Hu W. P., Wang C. R., Li Y. F., Yang S. H. (2011). Chem.–Eur. J..

[cit24] Lee G., Shim J. H., Kang H., Nam K. M., Song H., Park J. T. (2009). Chem. Commun..

[cit25] Vinodgopal K., Haria M., Meisel D., Kamat P. (2004). Nano Lett..

[cit26] Zhang Q., Bai Z. Y., Shi M., Yang L., Qiao J. L., Jiang K. (2015). Electrochim. Acta.

[cit27] Barzegar H. R., Hu G. Z., Larsen C., Jia X., Edman L., Wågberg T. (2014). Carbon.

[cit28] Hollamby M. J., Karny M., Bomans P. H. H., Sommerdijk N. A. J. M., Saeki A., Seki S., Minamikawa H., Grillo I., Pauw B. R., Brown P., Eastoe J., Möhwald H., Nakanishi T. (2014). Nat. Chem..

[cit29] Michinobu T., Nakanishi T., Hill J. P., Funahashi M., Ariga K. (2006). J. Am. Chem. Soc..

[cit30] Duer M. J. , Solid State NMR Spectroscopy: Principles and Applications, John Wiley & Sons, 2002

[cit31] Marchetti A., Chen J. E., Pang Z. F., Li S. H., Ling D. S., Deng F., Kong X. Q. (2017). Adv. Mater..

