# Supplementary material for: Self-assembly of highly conductive self-n-doped fullerene ammonium halides and their application in the in situ solution-processable fabrication of working electrodes for alcohol electrooxidation
Source: RSC Adv. 2018 Mar 6;8(17):9503–11. doi: 10.1039/c8ra00100f (PMC9078672; doi:10.1039/c8ra00100f)
Supplement: RA-008-C8RA00100F-s001 [file RA-008-C8RA00100F-s001.pdf]

Electronic Supplementary Material (ESI) for RSC Advances.
This journal is © The Royal Society of Chemistry 2018

Supporting Information

Self-assembly of highly conductive self-*n*-doped fullerene ammonium halides and their application in the in situ solution-processable fabrication of working electrodes for alcohol electrooxidation

Huanhuan Wang†, Xuan Sun†, Zhouchen Lin, Zhenfeng Pang, Xueqian Kong, Ming Lei* and Yongfang Li

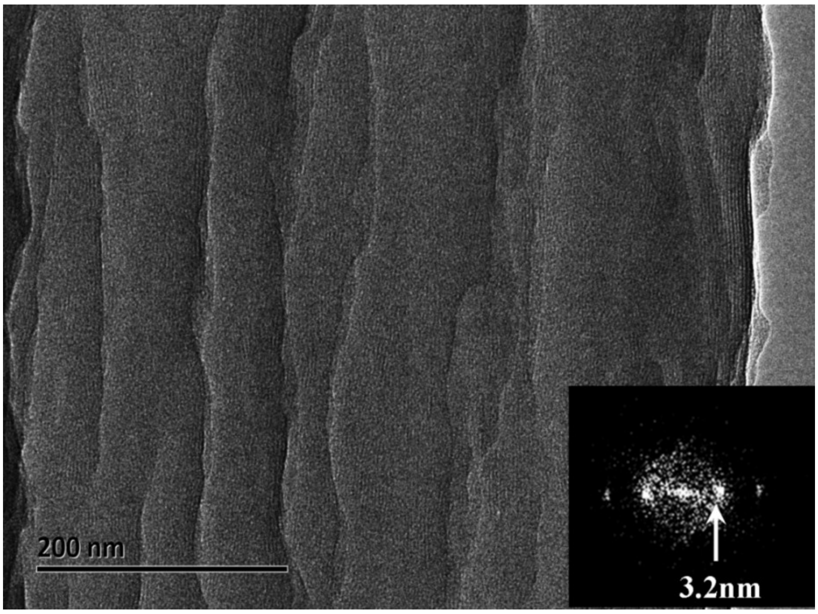

Figure S1. Cross-sectional high resolution TEM image of large area self-assembled PCBDANI film.
